# Spirituality, Religious Attendance and Health Complaints in Czech Adolescents

**DOI:** 10.3390/ijerph17072339

**Published:** 2020-03-30

**Authors:** Radka Zidkova, Petr Glogar, Iva Polackova Solcova, Jitse P. van Dijk, Michal Kalman, Peter Tavel, Klara Malinakova

**Affiliations:** 1 Olomouc University Social Health Institute, Palacky University Olomouc, 771 11 Olomouc, Czech; petr.glogar@oushi.upol.cz (P.G.); j.p.van.dijk@umcg.nl (J.P.v.D.); peter.tavel@oushi.upol.cz (P.T.); klara.malinakova@oushi.upol.cz (K.M.); 2 Institute of Psychology, Czech Academy of Science, 11000 Prague, Czech; polackova@praha.psu.cas.cz; 3 Department of Community and Occupational Medicine, University Medical Center Groningen, University of Groningen, 9713 AV Groningen, The Netherlands; 4 Graduate School Kosice Institute for Society and Health, P.J. Safarik University in Kosice, 040 11 Kosice, Slovak; 5 Department of Recreation and Leisure Studies, Faculty of Physical Culture, Palacky University in Olomouc, 771 11 Olomouc, Czech; michal.kalman@upol.cz

**Keywords:** health complaints, psychosomatic syndrome, adolescents, religiosity, spirituality, secular environment

## Abstract

Research in some religious countries shows that religiosity and spirituality positively affect adolescent health. We studied whether religiosity and spirituality also have positive associations with adolescent health in a secular country. We tested the associations between religious attendance and spirituality and self-reported health and health complaints using a representative sample of Czech adolescents (*n* = 4182, 14.4 ± 1.1 years, 48.6% boys) from the 2014 health behavior in school-aged children (HBSC) study. We used religious attendance, the adjusted shortened version of the spiritual well-being scale (SWBS), and its two components—religious well-being (RWB) and existential well-being (EWB)—as independent variables and the eight item “HBSC symptom checklist” and self-reported overall health as dependent variables. A higher level of spirituality was associated with lower chances of health complaints and self-reported health, ranging from a 9% to 30% decrease in odd ratios (OR). Religious attendance was not associated with any of the observed variables. The EWB showed a negative association with all of the observed variables, with associations ranging from a 19% to 47% decrease. The RWB was associated with a higher risk of nervousness (OR = 1.12), while other associations were not significant. Non-spiritual but attending respondents were more likely to report a higher occurrence of stomachache (OR = 2.20) and had significantly worse overall health (OR = 2.38). In a largely secular country, we found that spirituality and the EWB (unlike religious attendance and the RWB) could have a significant influence on adolescent health.

## 1. Introduction

Adolescence is a dynamic transitional process accompanied by physical, psychological, and emotional changes [1] associated with brain maturation, endocrine change, and physical growth. These changes are very complex and their effect on health and well-being are profound. Adolescence marks a transition in risks for depression and other mental disorders, substance misuse, and antisocial behavior, as well as a fast-track to adulthood with early transitions into sexual activity and school leaving and psychosomatic syndromes [2]. Several common pain syndromes, such as headache, stomachache, back and facial ache, begin or get worse in early adolescence, and these pains more often coexist than occur in isolation [3,4].

However, the incidence of somatic symptoms is associated not only with biological and psychological changes but also with a range of other factors, such as physical activity [5,6,7]. They are also associated with psycho-social determinants of health, e.g., relationships with parents and the family social-economic background [8]. In addition, there are studies that have found a relation between religiosity and spirituality (R/S) and fewer somatic symptoms or that an R/S intervention reduced somatic symptoms [9,10,11]. Although most of the research has examined a relationship between R/S and health, there are also some studies examining the potential for translation into clinical practice [12]. This provides a strong argument for studying adolescents’ health and well-being in a broader context and further exploring a possible positive role of R/S in adolescent health complaints.

Spirituality is an internal system of beliefs that includes a connection to what an individual feels to be sacred and transcendent and in a broader sense typically involves a search for meaning and purpose in life [9]. Religiosity involves beliefs, practices, and rituals related to the transcendent, i.e., a higher power. Religions usually have specific beliefs about life after death and rules about how to behave within a social group [9].

Research suggests that religiosity and spirituality have positive effects on adolescent health attitudes or behaviors [13]. Spirituality (distinctly and in relation to religiosity) has been shown to be associated with the healthy development of adolescents [14] and their lower health-risk behavior [15]. It has further noted their more active ways of spending leisure time [16], with an enhanced ability to cope and with positive outcomes in mental health, psychological well-being, and academic learning [14]. Both spirituality and religiosity are usually associated with better mental health as well [17,18,19].

However, research reporting positive associations of adolescent R/S with their health has mostly been conducted in countries with a religious background. We have not found any research investigating the relationship between R/S and health in secular countries, though studies reporting on the relationship between religiosity and suicidality in a secular country did exist [20,21]. Therefore, it is still not clear whether these results could also be generalized to a secular environment. The Czech Republic is one of the most secular countries in the world, with nearly three-quarters of adults (70.5%) describing themselves as non-religious or even atheist [22] and roughly two-thirds (66%) declaring that they do not believe in God [23].

Thus, it is possible that these specific conditions might influence the observed relationships. Therefore, the aim of this study is to explore the associations of spirituality, religiosity and adolescent health complaints in a secular environment.

## 2. Materials and Methods

### 2.1. Participants and Procedure

We used data based on a nationally representative sample of Czech boys and girls from the 2014 health behavior in school-aged children (HBSC) study. This international cross-sectional WHO study is focused on the well-being, health, health-related behavior and their gender and socioeconomic determinants in 11-, 13-, and 15-year-old adolescents. The HBSC study has been conducted since 1983/84 in four-year intervals and now covers 49 countries across Europe and North America. According to the HBSC study protocol, schools were selected randomly after stratification by region, school type (primary schools vs. secondary schools), and school size, ensuring that the sample is representative. Out of 243 contacted schools 242 schools agreed to participate (response rate 99.6%). Then, classes from the 5th, 7th, and 9th grades, in general corresponding to the age categories of 11-, 13-, and 15-year-olds, were selected at random, one from each grade per school.

Data from 14,539 Czech pupils were obtained (response rate 89.2%). The reason for non-response was mostly illness or other reasons, e.g., academic competitions or sports (10.6%), and 30 children (0.2%) refused to participate in the survey. There were two versions of the survey. The spirituality questionnaire was involved in only one of them and in addition was distributed only to participants of the 7th and 9th grades. The spirituality questionnaire was not distributed to participants of the 5th grades. Thus, for the purpose of this study, the dataset contained 4889 adolescents. Due to incomplete information on gender and age (we decided to include only participants between 12.5 and 16.4 of age) and spirituality or religiosity, 707 questionnaires were excluded. The final sample therefore contained 4182 respondents (mean age= 14.43, SD = 1.07, 48.6% boys).

Data were collected between April and June 2014. The questionnaires were distributed by trained administrators in the absence of classroom teachers in order to reduce response bias. Respondents had one school lesson (45 min) to complete the questionnaire. Participation in the survey was anonymous.

The study design was approved by the Ethics Committee of the Faculty of Physical Culture, Palacky University in Olomouc (No. 17/2013).

### 2.2. Measures

Health complaints were measured using a standard non-clinical measure of subjective health, also referred as “the HBSC symptom checklist” or “psychosomatic symptoms”. It explores the prevalence of eight symptoms: (1) headache, (2) stomachache, (3) backache, (4) feeling low, (5) irritability or bad temper, (6) feeling nervous, (7) sleeping difficulties, and (8) feeling dizzy. For each item, the respondents were asked how often they had experienced the problem in the last 6 months. They answered on a five-point scale that ranged from “every day” (1) to “rarely or never” (5). For the purpose of further analysis, the answers for each item were dichotomized, so that answers 1–3 (about every day, more than once a week and about every week) were considered to mean the symptom was prevalent.

Self-reported health was measured by the question: “Would you say your health is…?” Response options were excellent, good, fair, and poor.

Religious attendance was measured by the question: “How often do you go to church or to religious sessions?” with possible answers: (1) several times a week, (2) approximately once a week, (3) approximately once a month, (4) a few times a year, (5) exceptionally, and (6) never. Sunday attendance is a matter of obligation in most of the churches/denominations in the Czech Republic; therefore, participants who reported attending religious sessions at least once a week were dichotomized as attending.

Spirituality was measured using the adjusted shortened version [24] of the spiritual well-being scale (SWBS) [25]. The adjusted scale measures overall spiritual well-being and consists of seven items. Four items form the religious well-being subscale (RWB) and provide a self-assessment of one’s relationship to God (e.g., “I have a personally meaningful relationship with God.”). Three items form the existential well-being subscale (EWB), with items focusing on hope for the future and meaning in life (e.g., “I believe there is some real purpose for my life.”). Response options for each item consisted of a six-point Likert scale ranging from (1) strongly disagree to (6) strongly agree. The overall score from the adjusted shortened SWBS is computed by summing the responses to all seven items and ranges from 7 to 42, with a higher score representing greater spiritual well-being. In the analyses, spirituality was used as a continuous variable, but for the purpose of dichotomization for a sensitivity analysis, participants with a score of 34 or higher (upper quartile of the score) were considered as spiritual, and the rest as non-spiritual. Cronbach’s alpha was 0.81 in our sample.

### 2.3. Statistical Analyses

First, we performed descriptive analyses of the study sample. Because of the non-normal distribution of R/S data, we assessed the associations between religious attendance and spirituality and eight kinds of health complaints and self-reported health using binary logistic regression models adjusted for gender and age. Using the binary logistic regression approach avoids the normality assumption and provides easier interpretation of the results. In Model 1 we assessed the associations of religious attendance, and in Model 2 the associations of spirituality (SWBS) as well as the two SWBS dimensions (the EWB and RWB subscales). In Model 3 we assessed associations of a composite R/S variable (four combinations of religious attendance and dichotomized spirituality). We performed all analyses using the statistical software package IBM SPSS version 21 (IBM Corp., Armonk, NY, USA). 

## 3. Results

### 3.1. Description of the Population

Table 1 presents the background characteristics of the sample and describes the prevalence of eight kinds of health complaints and self-reported bad health. Of the 4182 respondents, 296 (7.1%) reported attending church services once a week or more and 399 (9.5%) were spiritual. The average spirituality score (SWBS) in the whole sample was 22.0. Religious attendance and spirituality were moderately correlated (r = 0.30).

### 3.2. Association of R/S and Adolescent Health Complaints

Table 2 shows the associations of religious attendance, spirituality, and their combination with eight kinds of health complaints and self-rated health, adjusted for gender and age. Mere religious attendance (Model 1) was not significantly associated with any of the observed variables, while a higher level of spirituality (Model 2) was associated with lower chances of health complaints in seven of the eight observed variables, the exception being nervousness, with associations ranging from a 9% decrease in odd ratios (OR) of the prevalence of stomachache (*p* < 0.05) to a 30% decrease in the odds of overall self-reported health (*p* < 0.001). When assessing separate subscales, EWB showed a negative association with all of the observed variables, with associations ranging from a 19% decrease in the odds of the prevalence of backache (*p* < 0.001) to a 47% decrease in the odds of overall self-reported good health (*p* < 0.001). On the other hand, RWB was associated with a higher risk of nervousness (OR = 1.12, *p* < 0.001), while other associations were not significant.

## 4. Discussion

The aim of this article was to assess the relationship of religious attendance, spirituality, and adolescent health complaints and self-rated health in a secular environment. The results showed no associations for religious attendance; however, attending non-spiritual adolescents were more likely to report stomachache and worse self-rated health. In contrast, we found that a higher level of spirituality (the SWBS scale) was associated with lower chances of health complaints (with the exception of nervousness). When the two dimensions of SWBS were observed separately, religious well-being showed significant associations with adolescents’ nervousness, while existential well-being was associated with a lower risk of all the health complaints.

Our first observation was that religious attendance of Czech adolescents had no association with their health complaints, which is in contrast to the findings of other authors, who reported that religious participation affects health outcomes [26,27]. An explanation of these discrepancies could be the different religious backgrounds. While other studies were conducted in a religious environment, our study was performed in one of the most secular countries in the world. This claim is supported by a previous study [28], which suggested that the relation of religiosity with self-rated health largely depends on the regional level of religiosity. In countries in which religiosity represents a social norm (i.e., it is ordinary and socially desirable), religious individuals report better subjective health than nonreligious individuals.

We further found that attending non-spiritual respondents had significantly higher chances of suffering from stomachache and had worse self-reported health. This seems to suggest that if adolescents attend religious services without being spiritual (or in other words without having a relationship to a higher entity), they are probably attending because they are only fulfilling parental expectations rather than following their own inner convictions. Especially in a secular environment, this situation could be difficult for adolescents surrounded by their non-religious peers. This explanation is also supported by other studies on R/S in Czech adolescents, which reported that attending non-spiritual adolescents showed a higher health-risk behavior [15] and that religious attendance was significantly associated with more difficult communication with the mother [29]. In addition, other studies have reported possible damaging effects of family arguments about religion on a child’s development [30]. Consequently, problems with internal insecurity, adaptation to a secular environment (non-religious peers) and quality of parent-adolescent relationships can affect adolescents’ psychological well-being and related somatic symptoms. However, this requires further analysis to find a causal pathway.

Findings on associations of spirituality with lower risk of health complaints, as we found in our study, are consistent with those of other authors, who suggest that spirituality is related to positive outcomes in mental health and psychological well-being and that it promotes healthy development [9,14,31]. Some authors argue that religiosity is more related to behavioral norms and is therefore associated with better health behavior, while spirituality is more connected with one’s own personal experience and could therefore be more strongly linked to physiological variables (e.g., cardiac reactivity and blood pressures) [32]. Therefore, spiritual adolescents could have fewer health complaints. An exception in the study was nervousness, where we found no significant association with spirituality. The reason for this exception is evident after the division of the SWBS into two separate components—the RWB and the EWB. The RWB was associated with a higher risk of nervousness, while the EWB was related to a lower risk of nervousness. As a result, the individual influences can be blurred.

We ultimately found that the RWB was associated with more frequent nervousness, while the EWB was clearly associated with a lower risk of all observed variables and better self-rated health. This is in contrast to earlier results, which showed that people who have a spiritual understanding of life in the absence of a religious framework are vulnerable to a mental disorder [33]. Research showed that for younger adolescents God has mostly anthropomorphic attributes, and only from 14 years onward (together with evolvement of abstract thinking) do adolescents report more abstract and relational conceptions of God [34]. Therefore, it is possible that many of our respondents have not yet accepted and internalized their religiosity, i.e., they do not see God (or another higher entity) as a source of help, meaning and hope in life leading to their resilience. The higher associations of nervousness with the RWB could be a consequence of the secular environment, as we described above. It is also possible that adolescents with some problems (physical or mental) are more likely to seek help in religiosity (relationship with God), while at the same time they may perceive less meaning in their lives and less hope for the future, which can also be an explanation for lower EWB values.

### 4.1. Strengths and Limitations

The strengths of this study are its large and representative sample and its high response rate, together with the use of the well-established HBSC methodology. To the best of our knowledge, this is one of the first studies examining the association of adolescent religiosity, religious attendance, spirituality, and health complaints in a secular environment. Some limitations should be mentioned as well. One limitation of this study is the high proportion of religiously non-affiliated respondents and the appropriately low number of religious respondents, because this decreases the power of the study. The next limitation is that church attendance is only a part of the religiosity and therefore it is not a precise measure of religion. A further limitation may be information bias, as our data were based on adolescents’ self-assessment, which may be influenced by a social suitability. Another limitation may be group heterogeneity due to various levels of internalization and acceptance of religiosity among adolescent. Finally, our design does not allow us to come to conclusions on causality.

### 4.2. Implications

Our findings suggest that the spirituality of adolescents may affect their health and therefore it needs to be nurtured. Such nurturing could concern, e.g., activities promoting the process of adolescents finding their own identity and healthy spirituality. We also found, in contrast, that religious attendance without spirituality could increase the risk of worse self-reported health. Therefore, it might be useful to inform parents about religious education in families and problems related to the discrepancy between one’s religiosity and spirituality level. Our results support the findings which suggest that it would be more appropriate to support a healthy internalization of spiritual values. Our results also show that future research on the associations of adolescents’ health-complaints and R/S should distinguish between these two concepts and, if possible, assess both dimensions.

## 5. Conclusions

Our findings suggest that in the Czech Republic, religious attendance and religious well-being for the most part do not have any impact on adolescent health complaints. However, religious attendance without spirituality was associated with worse self-reported health and that spirituality and existential well-being could have a significant influence on adolescent health. This means that in the secular Czech environment it is adolescents’ hope and meaning of life rather than a concept of “God” that is positively linked to their health.

## Figures and Tables

**Table 1:** Characteristics of the sample.

Variables	Total		Religious Attendance				Spirituality			
			Attending		Non-Attending		Spiritual		Non-Spiritual	
	*n*	%	*n*	%	*n*	%	*n*	%	*n*	%
Gender										
Boys	2034	48.6	131	44.3	1903	49.0	213	53.4	1821	48.1
Girls	2148	51.4	165	55.7	1983	51.0	186	46.6	1962	51.9
Age										
13 years (7th grade)	2091	50.0	146	49.3	1945	50.1	245	61.4	1846	48.8
15 years (9th grade)	2091	50.0	150	50.7	1941	49.9	152	38.6	1937	51.2
Health complaints										
Headache	1232	29.6	99	33.4	1133	29.3	105	26.5	1127	29.6
Stomachache	611	14.7	54	18.4	557	14.4	56	14.2	555	14.7
Backache	1118	27.0	80	27.3	1038	26.9	105	26.5	1118	27.0
Feeling low	1361	32.9	104	35.6	1257	32.6	124	31.4	1237	33.0
Irritability	2104	50.7	153	51.9	1951	50.6	194	48.9	1910	50.9
Nervousness	2214	53.3	169	57.5	2045	52.9	228	57.6	1986	52.8
Sleeping difficulties	1417	34.1	97	32.8	1320	34.2	119	30.1	1298	34.5
Dizziness	363	8.7	28	9.5	335	8.7	39	9.8	324	8.6
Self-reported bad health ^a^	716	17.1	53	17.9	663	17.1	54	13.5	662	17.6
Total	4182	100	296	7.1	3886	92.9	399	9.5	3783	90.5

Notes: Number of missing cases per variable: religious attendance—0; headache —16; stomachache—24; backache—36; feeling low—39; irritability—30; nervousness—25; sleeping difficulties—28; dizziness—19; self-reported bad health—17. ^a^ Only numbers regarding respondents with the occurrence of health-complaints or self-reported bad health are presented.

**Table 2:** Associations of adolescent health complaints with religious attendance, spirituality, and their combination, adjusted for age and gender (odds ratios (OR) and 95% confidence intervals, (CI)). Spiritual well-being scale: SWBS; religious well-being: RWB; existential well-being: EWB.

Caption	Headache	Stomachache	Backache	Feeling Low	Irritability	Nervousness	Sleeping Difficulties	Dizziness	Self-Reported Health
**Model 1: Religious Attendance**									
Attendance vs. Non-attendance	1.17 (0.91–1.52)	1.30 (0.95–1.78)	0.96 (0.76–1.30)	1.01 (0.85–1.42)	1.03 (0.81–1.31)	1.18 (0.93–1.50)	1.07 (0.71–1.61)	1.07 (0.71–1.61)	1.04 (0.76–1.41)
**Model 2: Spirituality (per SD)**									
SWBS-total	0.84 (0.79–0.90) ***	0.91 (0.83–0.99) *	0.90 (0.84–0.97) **	0.84 (0.78–0.90) ***	0.84 (0.79–0.89) ***	0.97 (0.91–1.03)	0.86 (0.81–0.92) ***	0.88 (0.79–0.99) *	0.70 (0.64–0.76) ***
RWB	0.97 (0.91–1.04)	1.07 (0.98–1.16)	1.01 (0.94–1.08)	1.04 (0.97–1.11)	0.99 (0.94–1.06)	1.12 (1.05–1.19) ***	1.01 (0.94–1.07)	1.06 (0.95–1.18)	0.95 (0.87–1.03)
EWB	0.75 (0.70–0.81) ***	0.76 (0.70–0.83) ***	0.81 (0.75–0.86) ***	0.67 (0.63–0.72) ***	0.70 (0.65–0.74) ***	0.78 (0.73–0.83) ***	0.74 (0.70–0.79) ***	0.73 (0.66–0.81) ***	0.53 (0.40–0.63) ***
**Model 3: Religious Attendance and spirituality (dichotomised) combined**									
Attending spiritual	1	1	1	1	1	1	1	1	1
Attending non-spiritual	1.61 (0.97–2.64)	2.20 (1.18–4.01) *	1.21 (0.72–2.02)	1.55 (0.94–2.54)	1.51 (0.95–2.40)	0.88 (0.55–1.40)	1.43 (0.88–2.35)	1.04 (0.47–2.27)	2.38 (1.27–4.44) **
Non-attending Spiritual	1.00 (0.62–1.59)	1.38 (0.76–2.52)	1.22 (0.77–1.94)	1.23 (0.78–1.93)	1.25 (0.83–0.88)	0.97 (0.64–1.47)	1.21 (0.77–1.89)	1.24 (0.62–2.48)	1.37 (0.74–2.51)
Non-attending Non-spiritual	1.10 (0.76–1.58)	1.18 (0.72–1.93)	1.10 (0.75–1.60	1.13 (0.78–1.62)	1.18 (0.85–1.64)	0.79 (0.56–1.09)	1.32 (0.92–1.89)	0.93 (0.53–1.64)	1.58 (0.96–2.60)

Notes: * *p* < 0.05, ** *p* < 0.01, *** *p* < 0.001. Those with *p*-values below 0.05 are considered significant and are shown in bold.
